# Crystal structure of Li_3_Ga(BO_3_)_2_

**DOI:** 10.1107/S205698901700295X

**Published:** 2017-02-28

**Authors:** Robert W. Smith, Darien Holman, Eric M. Villa

**Affiliations:** a Department of Chemistry, University of Nebraska at Omaha, 6001 Dodge Street, Omaha, Nebraska 68182, USA; b Department of Chemistry, Creighton University, 2500 California Plaza, Omaha, Nebraska 68178, USA

**Keywords:** crystal structure, crystal diffraction, gallium, borate

## Abstract

The crystal structure of trilithium gallium bis­(orthoborate), Li_3_Ga(BO_3_)_2_, is isotypic with Li_3_Al(BO_3_)_2_ in a triclinic cell in space-group type *P*-1. The three Li and the unique Ga atom are coordinated by four O atoms each in tetra­hedra, and the two B atoms are coordinated by three O atoms in orthoborate triangles. Chains with composition [Ga_2_(BO_3_)_4_]^6−^ extend along the *a* axis. The Li atoms inter­leave these chains in tetra­hedral inter­stices. A comparison is made between the structure model of the title compound and that of a previously reported model for a compound with the same composition [Abdullaev & Mamedov (1972 ▸). *Zh. Strukt. Khim*. **13**, 943–946.]

## Abstract 2

The title substance of lithium gallium borate, Li_3_Ga(BO_3_)_2_, crystallizes in a triclinic cell and is isotypic with the aluminium analog. The structure is composed of lithium- and gallium-centered tetra­hedra and boron-centered triangles.

## Chemical context   

**Figure 1:**
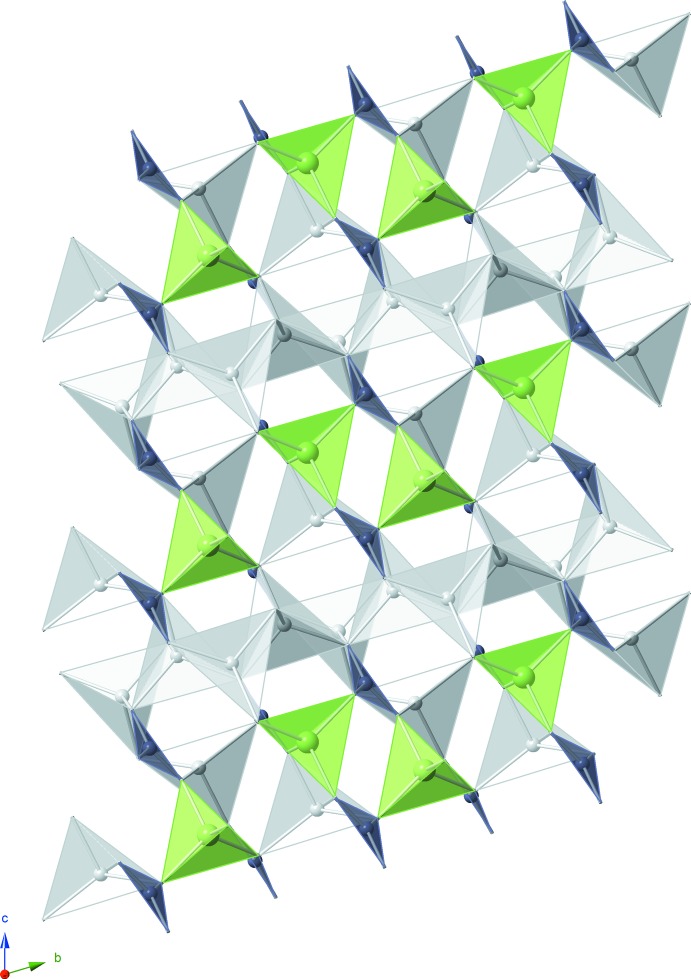
A projection of the crystal structure of Li_3_Ga(BO_3_)_2_ along the *a* axis. Infinite [Ga_2_(BO_3_)_4_]^6−^ chains are linked together by sheets of Li atoms in tetra­hedral voids. GaO_4_ tetra­hedra are light green, LiO_4_ tetra­hedra are light gray, and BO_3_ triangles are blue–grey. Corner O atoms have been omitted for clarity.

We are examining the alkali metal/gallium/borate phase diagrams, investigations of which have revealed to date, among others, the homologous series *A*_2_Ga_2_O(BO_3_)_2_, in which *A* = Na, K, Rb, and Cs (Corbel & Leblanc, 2000 ▸; Smith, 1995 ▸; Smith *et al.*, 1997 ▸,2008 ▸, respectively) and the homologous series *A*_3_Ga(BO_3_)_2_, in which *A* = Li, Na, K, Rb, and Cs. We report herein the crystal structure of the lithium analog (Fig. 1 ▸) of the latter series, which is the only one which melts congruently, which has a unique structure among the series, and which is isotypic with Li_3_Al(BO_3_)_2_ (He *et al.*, 2002 ▸); the other analogs have yet to be crystallized in the form of single crystals, but are structurally distinct from the lithium analog and isotypic with each other based on their powder X-ray diffraction patterns.

A crystal structure for this compound was previously reported by Abdullaev & Mamedov (1972 ▸) in the same triclinic space-group type *P* 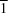, and with the same gallium-borate polyhedral pattern but with important differences with the structure reported herein, to wit: slightly different cell parameters and a different reduced cell, a significantly smaller cell volume (*i.e*., 3% smaller), less regular bond-valence sums (BVS), greater deviations from expected inter­atomic distances, and irregular, five- and six-coordinate lithium-centered polyhedra. Table 1 ▸ compares inter­atomic distances from the structure reported by Addullaev & Mamedov (1972) and this report, with expected distances using Shannon’s radii (Shannon, 1976 ▸); it also lists bond-valence sums for each structure. We have considered as bonds all Li—O distances under 3 Å from the 1972 report because doing so produces more reasonable BVS values, thus rendering some of the lithium atoms as being five- or six-coordinate in the previous structure report. It should be noted that the authors, however, reported all lithium atoms as tetra­hedrally coordinated. The present structure model clearly differs from the 1972 structure model and hence indicates a second possible modification for this composition. Whether a polymorphic relation exists between the two phases remains unknown and needs additional proof by using complementary methods such as thermal analysis.

## Structural commentary   

**Figure 2:**
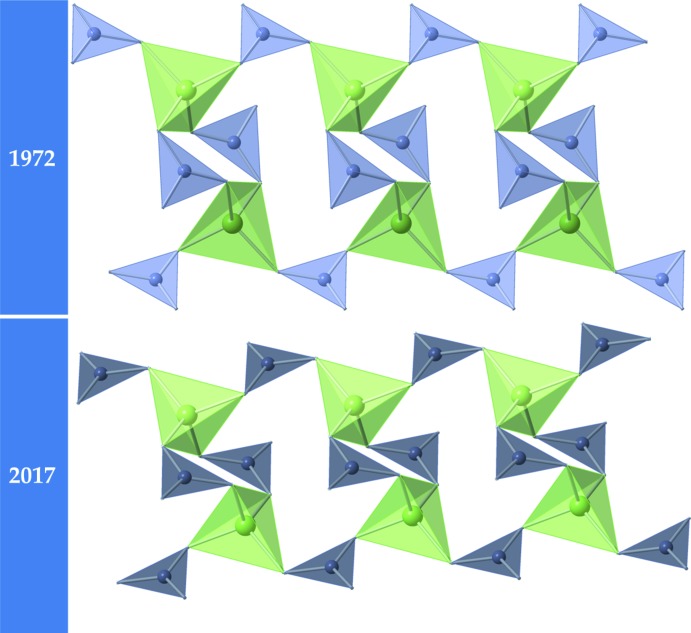
A comparison of the infinite [Ga_2_(BO_3_)_4_]^6−^ chains in the structures of Li_3_Ga(BO_3_)_2_ with respect to the model by Abdullaev & Mamedov (1972 ▸) (top) and the structure model presented in this manuscript. The connectivity of these two chains are the same but there are important differences in the actual bonding. In our structure model, these chains run down the *c* axis. Here the GaO_4_ tetra­hedra are light green and the BO_3_ triangles are blue–grey (which are shown in light blue–grey in the 1972 structure). Corner O atoms have been omitted for clarity.

The crystal structure of the title compound consists of lithium- and gallium-centered tetra­hedra and boron-centered triangles, all of which share oxygen vertices (Fig.1). Each GaO_4_ tetra­hedron is linked to four BO_3_ triangles and six LiO_4_ tetra­hedra. The gallium-centered tetra­hedra and boron-centered triangles adjoin through shared vertices to form infinite chains of composition [Ga_2_(BO_3_)_4_]^6−^, with the chains extending parallel to the *a* axis; lithium cations inter­leave the chains in tetra­hedral inter­stices. Fig. 2 ▸ shows a comparison of the gallium-borate chains in both the previously reported structure (Abdullaev & Mamedov, 1972 ▸) and the structure presented here. The two exhibit the same connectivity but have sizeable differences in bond lengths, bond angles and bond-valence-sum values (see Table 1 ▸). Averaged inter­atomic distances for the title structure are consistent with those determined from the ionic radii reported by Shannon (1976 ▸), *viz.* 1.97 (5), 1.85 (2), and 1.39 (4) Å for the experimentally determined Li—O, Ga—O, and B—O distances, respectively. We also calculated the bond-valence-um values for each element using the values provided by Brese & O’Keeffe (1991 ▸). The results (Table 1 ▸) are in good agreement with the expected values of 1, 3, 3 and 2 for Li, Ga, B and O atoms, respectively.

**Figure 3:**
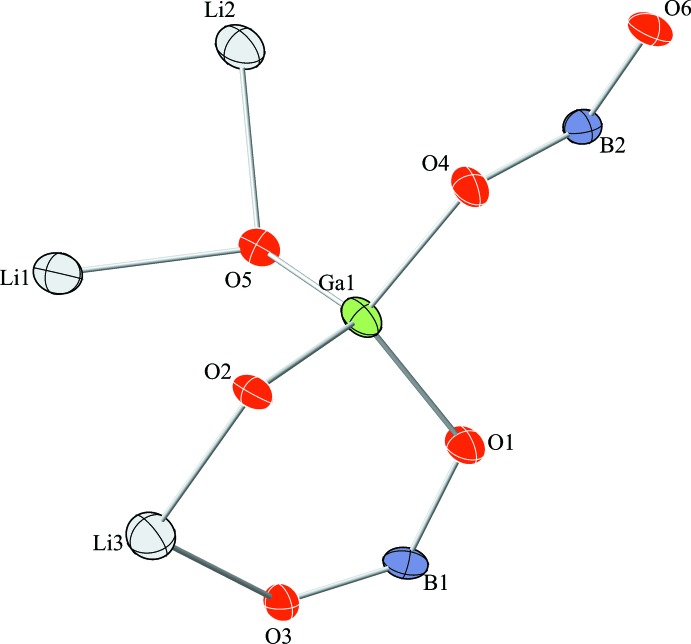
The asymmetric unit of the title structure with atom labelling. Displacement ellipsoids are drawn at the 75% probability level.

Lastly, Fig. 3 ▸ displays the anisotropic displacement parameters of the atoms within the asymmetric unit of the title structure.

## Synthesis and crystallization   

Powder samples were made by solid-state reactions starting with stoichiometric proportions of lithium nitrate, gallium(III) nitrate, and boric acid. We first ground the starting materials and fired them in an alumina crucible at 573 K for two h to decompose them to finely divided oxides, after which we progressively heated the samples to 973 K at 50 to 100 K and 24-hour increments, grinding the samples between each successive heat treatment. Samples were single-phase as revealed by powder X-ray diffraction.

Single crystals were grown from the melt. About 500 mg of sample were placed in a platinum dish, heated to 1033 K in a box oven, slow-cooled at 10 K h^−1^ to about 470 K, and then air-quenched. Several small, clear, colorless crystals were physically removed from the platinum crucible and mounted on a goniometer for a preliminary scan in order to find one of suitable quality.

## Refinement   

Crystal data, data collection and structure refinement details are summarized in Table 2 ▸.

## Supplementary Material

Crystal structure: contains datablock(s) I. DOI: 10.1107/S205698901700295X/wm5356sup1.cif

Structure factors: contains datablock(s) I. DOI: 10.1107/S205698901700295X/wm5356Isup3.hkl

Supporting information file. DOI: 10.1107/S205698901700295X/wm5356Isup3.cml

CCDC reference: 1534100

Additional supporting information:  crystallographic information; 3D view; checkCIF report

## Figures and Tables

**Table 1:** Comparison of the two structures with composition Li_3_Ga(BO_3_)_2_

Structure model	Abdullaev & Mamedov (1972 ▸)	Current work	Shannon (1976 ▸)
Reduced cell (Å, °)	4.90 (2) 6.23 (3) 7.78 (5) 72.9 (5) 90.0 (5) 90.0 (5)	4.8731 (3) 6.2429 (4) 8.0130 (5) 73.346 (6) 89.701 (5) 89.698 (5)	
			
Range of inter­atomic distances (Å)			
Li—O	2.28±0.41	1.965±0.054	1.97
Ga—O	2.07±0.43	1.847±0.021	1.85
B—O	1.31±0.13	1.384±0.038	1.39
			
Bond-valence-sum values and coordination numbers (in brackets)			
Li (1, 2 & 3)	1.14 [4 + 1], 1.01 [4], 1.00 [6]	1.07 [4], 1.03 [4], 1.05 [4]	
Ga (1)	2.38 [4]	2.92 [4]	
B (1 & 2)	3.17 [3], 3.06 [3]	2.91 [3], 2.91 [3]	

**Table 2:** Experimental details

Crystal data	
Chemical formula	Li_3_Ga(BO_3_)_2_
*M*_r_	208.16
Crystal system, space group	Triclinic, *P* 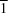
Temperature (K)	293
*a*, *b*, *c* (Å)	4.8731 (3), 6.2429 (4), 8.0130 (5)
α, β, γ (°)	73.346 (6), 89.701 (5), 89.698 (5)
*V* (Å^3^)	233.54 (3)
*Z*	2
Radiation type	Mo *K*α
μ (mm^−1^)	5.84
Crystal size (mm)	0.09 × 0.03 × 0.01

Data collection	
Diffractometer	Rigaku SCX mini diffractometer
Absorption correction	Multi-scan (*CrysAlis PRO*; Rigaku Oxford Diffraction, 2015 ▸)
*T*_min_, *T*_max_	0.785, 1.000
No. of measured, independent and observed [*I* > 2σ(*I*)] reflections	2936, 1397, 1265
*R*_int_	0.029
(sin θ/λ)_max_ (Å^−1^)	0.714

Refinement	
*R*[*F* ^2^ > 2σ(*F* ^2^)], *wR*(*F* ^2^), *S*	0.028, 0.067, 1.09
No. of reflections	1397
No. of parameters	109
Δρ_max_, Δρ_min_ (e Å^−3^)	1.04, −0.76

Computer programs: *CrysAlis PRO* (Rigaku Oxford Diffraction, 2015 ▸), *SHELXT* (Sheldrick, 2015*a* ▸), *SHELXL2014* (Sheldrick, 2015*b* ▸) and *OLEX2* (Dolomanov *et al.*, 2009 ▸).

## supplementary crystallographic information

### Crystal data

**Table:**

Li_3_Ga(BO_3_)_2_	*Z* = 2
*M**_r_* = 208.16	*F*(000) = 196
Triclinic, *P*1	*D*_x_ = 2.960 Mg m^−^^3^
*a* = 4.8731 (3) Å	Mo *K*α radiation, λ = 0.71073 Å
*b* = 6.2429 (4) Å	Cell parameters from 1687 reflections
*c* = 8.0130 (5) Å	θ = 2.7–32.5°
α = 73.346 (6)°	µ = 5.84 mm^−^^1^
β = 89.701 (5)°	*T* = 293 K
γ = 89.698 (5)°	Plate, clear light colourless
*V* = 233.54 (3) Å^3^	0.09 × 0.03 × 0.01 mm

### Data collection

**Table:**

Rigaku SCX mini diffractometer	1397 independent reflections
Radiation source: fine-focus sealed X-ray tube, Enhance (Mo) X-ray Source	1265 reflections with *I* > 2σ(*I*)
Graphite monochromator	*R*_int_ = 0.029
ω scans	θ_max_ = 30.5°, θ_min_ = 2.7°
Absorption correction: multi-scan (*CrysAlis PRO*; Rigaku Oxford Diffraction, 2015)	*h* = −6→6
*T*_min_ = 0.785, *T*_max_ = 1.000	*k* = −8→8
2936 measured reflections	*l* = −11→11

### Refinement

**Table:**

Refinement on *F*^2^	0 restraints
Least-squares matrix: full	Primary atom site location: dual
*R*[*F*^2^ > 2σ(*F*^2^)] = 0.028	*w* = 1/[σ^2^(*F*_o_^2^) + (0.0282*P*)^2^ + 0.1042*P*] where *P* = (*F*_o_^2^ + 2*F*_c_^2^)/3
*wR*(*F*^2^) = 0.067	(Δ/σ)_max_ < 0.001
*S* = 1.09	Δρ_max_ = 1.04 e Å^−^^3^
1397 reflections	Δρ_min_ = −0.76 e Å^−^^3^
109 parameters	

### Special details

**Table:**

Geometry. All esds (except the esd in the dihedral angle between two l.s. planes) are estimated using the full covariance matrix. The cell esds are taken into account individually in the estimation of esds in distances, angles and torsion angles; correlations between esds in cell parameters are only used when they are defined by crystal symmetry. An approximate (isotropic) treatment of cell esds is used for estimating esds involving l.s. planes.
Refinement. Crystals of Li_3_Ga(BO_3_)_2_ were mounted on MiTeGen Microloop with non-drying immersion oil. The crystal was then optically aligned on the Rigaku SCX-Mini diffractometer using a digital camera. Initial matrix images were collected to determine the unit cell, validity and proper exposure time. Three hemispheres (where φ= 0.0, 120.0 and 240.0) of data were collected with each consisting 180 images each with 1.00° widths and a 1.00° step. The structure of Li_3_Ga(BO_3_)_2_ was refined using SHELXT (Sheldrick, 2015) Intrinsic Phasing and SHELXL (Sheldrick, 2015). Olex2 (Dolomanov *et al.*, 2009) was used as a graphical interface. Images of the above compound were made using CrystalMaker for Windows, version 9.2.8. The refinement proceeded without any incidents and without any need for modelling disorder or twinning or any constraints or restraints. Refinement of the structure was based on F^2^ against all reflections. The R-factor R is based on F^2^>2?(F^2^), but is not relevant to the choice of reflections for refinement; whereas the weighted R-factor wR and goodness of fit S are based on F^2^. The maximum electron denisty is 1.035 and is located 1.629 Å from Li(2), 1.960 Å from Li(1) and 2.169 Å from a different Li(1), which leads to nothing reasonable. All other maximum peaks are under 0.600.

### Fractional atomic coordinates and isotropic or equivalent isotropic displacement parameters (Å^2^)

**Table:**

	*x*	*y*	*z*	*U*_iso_*/*U*_eq_	
Ga1	0.65937 (6)	0.22951 (4)	0.60906 (4)	0.00968 (10)	
B1	0.1691 (6)	0.0135 (5)	0.7454 (4)	0.0088 (5)	
B2	0.6631 (6)	0.5014 (5)	0.2489 (4)	0.0094 (5)	
O1	0.3037 (4)	0.1391 (3)	0.5958 (2)	0.0116 (4)	
O2	−0.1202 (4)	−0.0028 (3)	0.7317 (2)	0.0101 (4)	
O3	0.2978 (4)	−0.0830 (3)	0.8955 (2)	0.0109 (4)	
O4	0.7953 (4)	0.3547 (3)	0.3900 (2)	0.0117 (4)	
O5	0.3833 (4)	0.5529 (3)	0.2727 (2)	0.0100 (4)	
O6	0.7921 (4)	0.5853 (3)	0.0967 (2)	0.0120 (4)	
Li1	0.1828 (10)	−0.3717 (8)	1.0414 (6)	0.0154 (10)	
Li2	1.1645 (10)	0.2738 (8)	0.3670 (6)	0.0143 (9)	
Li3	0.6839 (10)	0.8785 (8)	−0.0399 (6)	0.0159 (10)	

### Atomic displacement parameters (Å^2^)

**Table:**

	*U*^11^	*U*^22^	*U*^33^	*U*^12^	*U*^13^	*U*^23^
Ga1	0.00877 (15)	0.01034 (15)	0.00789 (15)	−0.00023 (10)	0.00130 (10)	0.00064 (10)
B1	0.0103 (13)	0.0052 (11)	0.0106 (12)	0.0008 (9)	0.0021 (10)	−0.0018 (10)
B2	0.0091 (13)	0.0088 (12)	0.0095 (12)	−0.0013 (10)	0.0002 (10)	−0.0013 (10)
O1	0.0098 (9)	0.0134 (9)	0.0090 (8)	−0.0028 (7)	0.0011 (7)	0.0012 (7)
O2	0.0068 (8)	0.0105 (8)	0.0098 (8)	0.0013 (6)	0.0002 (7)	0.0020 (7)
O3	0.0107 (9)	0.0104 (8)	0.0094 (8)	−0.0007 (7)	−0.0018 (7)	0.0008 (7)
O4	0.0110 (9)	0.0138 (9)	0.0066 (8)	0.0017 (7)	0.0012 (7)	0.0030 (7)
O5	0.0088 (9)	0.0102 (8)	0.0104 (8)	0.0008 (7)	0.0013 (7)	−0.0017 (7)
O6	0.0136 (9)	0.0114 (9)	0.0088 (8)	−0.0001 (7)	0.0029 (7)	0.0005 (7)
Li1	0.019 (2)	0.013 (2)	0.013 (2)	−0.0038 (18)	0.0013 (18)	−0.0023 (18)
Li2	0.012 (2)	0.014 (2)	0.013 (2)	−0.0009 (17)	0.0008 (17)	0.0009 (17)
Li3	0.013 (2)	0.017 (2)	0.015 (2)	−0.0014 (18)	0.0000 (18)	−0.0016 (18)

### Geometric parameters (Å, º)

**Table:**

Ga1—O1	1.8385 (18)	B1—O2	1.421 (3)
Ga1—O2^i^	1.8440 (18)	B1—O3	1.339 (3)
Ga1—O4	1.8272 (18)	B2—O4	1.393 (3)
Ga1—O5^ii^	1.8759 (18)	B2—O5	1.423 (3)
B1—O1	1.393 (3)	B2—O6	1.336 (3)
			
O1—Ga1—O2^i^	111.84 (8)	Li1^xii^—Li1—Li3^vii^	149.2 (3)
O1—Ga1—O5^ii^	102.08 (8)	Li1^xii^—Li1—Li3^ix^	72.2 (2)
O1—Ga1—Li1^iii^	116.15 (10)	Li2^ix^—Li1—Ga1^iii^	63.94 (14)
O1—Ga1—Li2^iv^	124.51 (10)	Li2^ix^—Li1—Li3^viii^	60.89 (16)
O1—Ga1—Li2^v^	35.98 (10)	Li2^ix^—Li1—Li3^ii^	120.2 (2)
O1—Ga1—Li2^vi^	86.55 (10)	Li3^ii^—Li1—Ga1^iii^	56.40 (12)
O1—Ga1—Li2	133.42 (11)	Li3^vii^—Li1—Ga1^iii^	74.07 (15)
O1—Ga1—Li3^vii^	86.54 (11)	Li3^viii^—Li1—Ga1^iii^	117.59 (17)
O2^i^—Ga1—O5^ii^	111.37 (8)	Li3^ix^—Li1—Ga1^iii^	97.77 (17)
O2^i^—Ga1—Li1^iii^	76.62 (10)	Li3^vii^—Li1—Li2^ix^	117.0 (2)
O2^i^—Ga1—Li2^iv^	115.22 (10)	Li3^ix^—Li1—Li2^ix^	114.0 (2)
O2^i^—Ga1—Li2^v^	135.30 (11)	Li3^ii^—Li1—Li3^viii^	156.5 (2)
O2^i^—Ga1—Li2^vi^	37.59 (10)	Li3^vii^—Li1—Li3^viii^	107.55 (19)
O2^i^—Ga1—Li2	77.78 (10)	Li3^ix^—Li1—Li3^viii^	129.60 (18)
O2^i^—Ga1—Li3^vii^	39.95 (11)	Li3^vii^—Li1—Li3^ii^	49.58 (19)
O4—Ga1—O1	109.76 (8)	Li3^ix^—Li1—Li3^ii^	73.05 (19)
O4—Ga1—O2^i^	110.94 (8)	Li3^ix^—Li1—Li3^vii^	116.4 (2)
O4—Ga1—O5^ii^	110.54 (8)	Ga1—Li2—Ga1^vi^	105.15 (13)
O4—Ga1—Li1^iii^	126.18 (10)	Ga1^i^—Li2—Ga1^iv^	70.59 (10)
O4—Ga1—Li2	34.37 (10)	Ga1—Li2—Ga1^iv^	95.24 (13)
O4—Ga1—Li2^vi^	96.01 (11)	Ga1^i^—Li2—Ga1	104.73 (14)
O4—Ga1—Li2^v^	74.92 (11)	Ga1^i^—Li2—Ga1^vi^	81.11 (11)
O4—Ga1—Li2^iv^	78.99 (10)	Ga1^vi^—Li2—Ga1^iv^	148.57 (16)
O4—Ga1—Li3^vii^	150.82 (11)	Ga1—Li2—Li3^xiii^	135.80 (19)
O5^ii^—Ga1—Li1^iii^	34.75 (10)	Ga1^i^—Li2—Li3^xiii^	110.84 (17)
O5^ii^—Ga1—Li2^iv^	33.37 (10)	B1^viii^—Li2—Ga1^i^	136.51 (18)
O5^ii^—Ga1—Li2^v^	106.86 (11)	B1^viii^—Li2—Ga1^vi^	57.25 (11)
O5^ii^—Ga1—Li2^vi^	146.78 (10)	B1^viii^—Li2—Ga1^iv^	152.84 (18)
O5^ii^—Ga1—Li2	116.90 (11)	B1^viii^—Li2—Ga1	77.51 (13)
O5^ii^—Ga1—Li3^vii^	88.33 (11)	B1^viii^—Li2—B2^i^	135.2 (2)
Li1^iii^—Ga1—Li2^iv^	52.55 (11)	B1^viii^—Li2—Li1^xi^	97.11 (18)
Li2^v^—Ga1—Li1^iii^	137.36 (13)	B1^viii^—Li2—Li3^xiii^	58.70 (14)
Li2—Ga1—Li1^iii^	110.42 (13)	B2^i^—Li2—Ga1	146.69 (19)
Li2^vi^—Ga1—Li1^iii^	112.82 (12)	B2^i^—Li2—Ga1^vi^	100.55 (15)
Li2^v^—Ga1—Li2	104.73 (14)	B2^i^—Li2—Ga1^i^	58.67 (11)
Li2—Ga1—Li2^vi^	74.85 (13)	B2^i^—Li2—Ga1^iv^	53.11 (10)
Li2^vi^—Ga1—Li2^iv^	148.57 (16)	B2^i^—Li2—Li1^xi^	59.61 (14)
Li2^v^—Ga1—Li2^iv^	109.41 (10)	B2^i^—Li2—Li3^xiii^	76.46 (16)
Li2^v^—Ga1—Li2^vi^	98.89 (11)	O1^i^—Li2—Ga1^vi^	72.19 (15)
Li2—Ga1—Li2^iv^	84.76 (13)	O1^i^—Li2—Ga1	75.30 (16)
Li3^vii^—Ga1—Li1^iii^	59.79 (12)	O1^i^—Li2—Ga1^iv^	90.70 (18)
Li3^vii^—Ga1—Li2^vi^	59.91 (13)	O1^i^—Li2—Ga1^i^	34.43 (10)
Li3^vii^—Ga1—Li2^v^	121.95 (13)	O1^i^—Li2—B1^viii^	112.1 (2)
Li3^vii^—Ga1—Li2	117.45 (13)	O1^i^—Li2—B2^i^	93.10 (19)
Li3^vii^—Ga1—Li2^iv^	112.26 (13)	O1^i^—Li2—O2^viii^	102.8 (2)
O1—B1—O2	115.8 (2)	O1^i^—Li2—O5^i^	105.5 (2)
O1—B1—Li2^viii^	104.8 (2)	O1^i^—Li2—Li1^xi^	149.9 (3)
O1—B1—Li3^ii^	113.6 (2)	O1^i^—Li2—Li3^xiii^	124.6 (2)
O2—B1—Li2^viii^	47.04 (15)	O2^viii^—Li2—Ga1^vi^	32.82 (9)
O2—B1—Li3^ii^	111.9 (2)	O2^viii^—Li2—Ga1	102.44 (18)
O3—B1—O1	123.3 (2)	O2^viii^—Li2—Ga1^iv^	159.9 (2)
O3—B1—O2	120.9 (2)	O2^viii^—Li2—Ga1^i^	113.13 (19)
O3—B1—Li2^viii^	113.91 (19)	O2^viii^—Li2—B1^viii^	30.07 (10)
O3—B1—Li3^ii^	42.00 (16)	O2^viii^—Li2—B2^i^	110.6 (2)
Li3^ii^—B1—Li2^viii^	141.61 (18)	O2^viii^—Li2—Li1^xi^	99.0 (2)
O4—B2—O5	117.1 (2)	O2^viii^—Li2—Li3^xiii^	39.36 (13)
O4—B2—Li1^viii^	106.0 (2)	O4—Li2—Ga1	33.11 (10)
O4—B2—Li2^v^	88.65 (18)	O4—Li2—Ga1^vi^	121.9 (2)
O4—B2—Li3	149.2 (2)	O4—Li2—Ga1^i^	131.9 (2)
O5—B2—Li1^viii^	119.8 (2)	O4—Li2—Ga1^iv^	87.97 (18)
O5—B2—Li2^v^	41.16 (15)	O4—Li2—B1^viii^	71.58 (17)
O5—B2—Li3	88.65 (18)	O4—Li2—B2^i^	136.5 (2)
O6—B2—O4	121.2 (2)	O4—Li2—O1^i^	107.7 (2)
O6—B2—O5	121.6 (2)	O4—Li2—O2^viii^	101.6 (2)
O6—B2—Li1^viii^	40.79 (15)	O4—Li2—O5^i^	108.5 (2)
O6—B2—Li2^v^	137.1 (2)	O4—Li2—Li1^xi^	87.7 (2)
O6—B2—Li3	37.74 (15)	O4—Li2—Li3^xiii^	117.1 (2)
Li1^viii^—B2—Li2^v^	105.56 (17)	O5^i^—Li2—Ga1^iv^	31.06 (10)
Li1^viii^—B2—Li3	70.99 (16)	O5^i^—Li2—Ga1^i^	74.16 (15)
Li3—B2—Li2^v^	122.03 (17)	O5^i^—Li2—Ga1^vi^	127.9 (2)
Ga1—O1—Li2^v^	109.59 (17)	O5^i^—Li2—Ga1	125.0 (2)
B1—O1—Ga1	120.01 (17)	O5^i^—Li2—B1^viii^	140.4 (2)
B1—O1—Li2^v^	129.9 (2)	O5^i^—Li2—B2^i^	27.93 (10)
Ga1^v^—O2—Li2^viii^	109.59 (15)	O5^i^—Li2—O2^viii^	129.2 (3)
Ga1^v^—O2—Li3^ix^	103.87 (16)	O5^i^—Li2—Li1^xi^	44.44 (15)
B1—O2—Ga1^v^	123.74 (16)	O5^i^—Li2—Li3^xiii^	90.06 (19)
B1—O2—Li2^viii^	102.9 (2)	Li1^xi^—Li2—Ga1^vi^	121.89 (19)
B1—O2—Li3^ix^	114.5 (2)	Li1^xi^—Li2—Ga1^i^	116.94 (18)
Li3^ix^—O2—Li2^viii^	99.6 (2)	Li1^xi^—Li2—Ga1	119.78 (18)
B1—O3—Li1	120.6 (2)	Li1^xi^—Li2—Ga1^iv^	63.51 (13)
B1—O3—Li3^vii^	133.1 (2)	Li1^xi^—Li2—Li3^xiii^	64.81 (16)
B1—O3—Li3^ii^	110.7 (2)	Li3^xiii^—Li2—Ga1^iv^	120.57 (17)
Li1—O3—Li3^ii^	108.2 (2)	Li3^xiii^—Li2—Ga1^vi^	57.29 (12)
Li1—O3—Li3^vii^	95.7 (2)	Ga1^x^—Li3—Li1^ii^	63.82 (13)
Li3^vii^—O3—Li3^ii^	81.0 (2)	Ga1^x^—Li3—Li2^xiii^	62.79 (13)
Ga1—O4—Li2	112.52 (17)	B1^ii^—Li3—Ga1^x^	121.35 (18)
B2—O4—Ga1	127.86 (17)	B1^ii^—Li3—B2	69.64 (14)
B2—O4—Li2	119.6 (2)	B1^ii^—Li3—Li1^ii^	57.56 (14)
Ga1^ii^—O5—Li1^x^	113.47 (16)	B1^ii^—Li3—Li1^x^	106.5 (2)
Ga1^ii^—O5—Li2^v^	115.56 (17)	B1^ii^—Li3—Li1^xi^	80.20 (17)
B2—O5—Ga1^ii^	113.05 (16)	B1^ii^—Li3—Li2^xiii^	150.9 (2)
B2—O5—Li1^x^	110.1 (2)	B2—Li3—Ga1^x^	168.9 (2)
B2—O5—Li2^v^	110.9 (2)	B2—Li3—Li1^x^	60.43 (15)
Li2^v^—O5—Li1^x^	92.0 (2)	B2—Li3—Li1^ii^	127.1 (2)
B2—O6—Li1^viii^	112.8 (2)	B2—Li3—Li1^xi^	64.26 (15)
B2—O6—Li1^xi^	130.9 (2)	B2—Li3—Li2^xiii^	108.51 (19)
B2—O6—Li3	117.0 (2)	O2^xi^—Li3—Ga1^x^	36.18 (10)
Li1^xi^—O6—Li1^viii^	83.6 (2)	O2^xi^—Li3—B1^ii^	122.9 (2)
Li3—O6—Li1^xi^	95.2 (2)	O2^xi^—Li3—B2	141.0 (2)
Li3—O6—Li1^viii^	112.6 (2)	O2^xi^—Li3—Li1^ii^	77.94 (18)
B2^viii^—Li1—Ga1^iii^	150.65 (19)	O2^xi^—Li3—Li1^x^	130.1 (2)
B2^viii^—Li1—Li2^ix^	115.6 (2)	O2^xi^—Li3—Li1^xi^	80.86 (19)
B2^viii^—Li1—Li3^ix^	107.8 (2)	O2^xi^—Li3—Li2^xiii^	41.01 (14)
B2^viii^—Li1—Li3^ii^	117.46 (19)	O2^xi^—Li3—Li3^xiv^	118.1 (3)
B2^viii^—Li1—Li3^vii^	81.65 (18)	O3^ii^—Li3—Ga1^x^	97.47 (18)
B2^viii^—Li1—Li3^viii^	54.76 (14)	O3^x^—Li3—Ga1^x^	73.34 (15)
O3—Li1—Ga1^iii^	90.74 (18)	O3^x^—Li3—B1^ii^	116.5 (2)
O3—Li1—B2^viii^	81.99 (18)	O3^ii^—Li3—B1^ii^	27.27 (10)
O3—Li1—O5^vii^	110.1 (2)	O3^x^—Li3—B2	101.3 (2)
O3—Li1—O6^viii^	108.0 (2)	O3^ii^—Li3—B2	92.9 (2)
O3—Li1—O6^ix^	117.7 (3)	O3^x^—Li3—O2^xi^	103.3 (2)
O3—Li1—Li1^xii^	125.5 (3)	O3^ii^—Li3—O2^xi^	112.5 (2)
O3—Li1—Li2^ix^	153.3 (3)	O3^x^—Li3—O3^ii^	99.0 (2)
O3—Li1—Li3^vii^	42.42 (15)	O3^ii^—Li3—Li1^x^	108.3 (2)
O3—Li1—Li3^ii^	36.20 (14)	O3^ii^—Li3—Li1^xi^	103.2 (2)
O3—Li1—Li3^viii^	133.3 (2)	O3^x^—Li3—Li1^x^	41.91 (14)
O3—Li1—Li3^ix^	75.85 (19)	O3^ii^—Li3—Li1^ii^	35.65 (13)
O5^vii^—Li1—Ga1^iii^	31.78 (9)	O3^x^—Li3—Li1^ii^	99.1 (2)
O5^vii^—Li1—B2^viii^	126.2 (2)	O3^x^—Li3—Li1^xi^	153.9 (3)
O5^vii^—Li1—Li1^xii^	124.5 (3)	O3^ii^—Li3—Li2^xiii^	153.3 (2)
O5^vii^—Li1—Li2^ix^	43.60 (14)	O3^x^—Li3—Li2^xiii^	92.5 (2)
O5^vii^—Li1—Li3^viii^	86.42 (18)	O3^ii^—Li3—Li3^xiv^	49.37 (18)
O5^vii^—Li1—Li3^ix^	126.0 (2)	O3^x^—Li3—Li3^xiv^	49.60 (17)
O5^vii^—Li1—Li3^ii^	82.63 (18)	O6—Li3—Ga1^x^	149.2 (2)
O5^vii^—Li1—Li3^vii^	76.30 (19)	O6—Li3—B1^ii^	82.26 (18)
O6^ix^—Li1—Ga1^iii^	95.39 (18)	O6—Li3—B2	25.26 (10)
O6^viii^—Li1—Ga1^iii^	149.8 (2)	O6—Li3—O2^xi^	115.8 (3)
O6^viii^—Li1—B2^viii^	26.37 (10)	O6—Li3—O3^ii^	109.1 (2)
O6^ix^—Li1—B2^viii^	113.2 (2)	O6—Li3—O3^x^	116.1 (3)
O6^viii^—Li1—O5^vii^	118.0 (2)	O6—Li3—Li1^x^	74.64 (19)
O6^ix^—Li1—O5^vii^	106.5 (2)	O6—Li3—Li1^xi^	42.94 (15)
O6^ix^—Li1—O6^viii^	96.4 (2)	O6—Li3—Li1^ii^	135.9 (2)
O6^viii^—Li1—Li1^xii^	48.01 (17)	O6—Li3—Li2^xiii^	86.9 (2)
O6^ix^—Li1—Li1^xii^	48.35 (17)	O6—Li3—Li3^xiv^	126.2 (3)
O6^viii^—Li1—Li2^ix^	92.4 (2)	Li1^x^—Li3—Ga1^x^	112.15 (18)
O6^ix^—Li1—Li2^ix^	75.35 (18)	Li1^xi^—Li3—Ga1^x^	116.62 (19)
O6^viii^—Li1—Li3^vii^	103.7 (2)	Li1^x^—Li3—Li1^ii^	130.42 (19)
O6^ix^—Li1—Li3^ii^	106.1 (2)	Li1^xi^—Li3—Li1^x^	116.4 (2)
O6^viii^—Li1—Li3^ii^	143.8 (2)	Li1^xi^—Li3—Li1^ii^	106.95 (18)
O6^ix^—Li1—Li3^vii^	155.5 (3)	Li1^x^—Li3—Li2^xiii^	96.3 (2)
O6^viii^—Li1—Li3^ix^	109.5 (2)	Li1^xi^—Li3—Li2^xiii^	73.57 (18)
O6^ix^—Li1—Li3^ix^	41.90 (15)	Li2^xiii^—Li3—Li1^ii^	118.79 (19)
O6^ix^—Li1—Li3^viii^	96.9 (2)	Li3^xiv^—Li3—Ga1^x^	83.1 (2)
O6^viii^—Li1—Li3^viii^	33.21 (13)	Li3^xiv^—Li3—B1^ii^	70.0 (2)
Li1^xii^—Li1—Ga1^iii^	136.1 (3)	Li3^xiv^—Li3—B2	100.9 (3)
Li1^xii^—Li1—B2^viii^	67.7 (2)	Li3^xiv^—Li3—Li1^xi^	150.0 (3)
Li1^xii^—Li1—Li2^ix^	81.0 (2)	Li3^xiv^—Li3—Li1^ii^	59.6 (2)
Li1^xii^—Li1—Li3^viii^	57.4 (2)	Li3^xiv^—Li3—Li1^x^	70.8 (2)
Li1^xii^—Li1—Li3^ii^	144.5 (3)	Li3^xiv^—Li3—Li2^xiii^	136.2 (3)
			
Ga1—O4—Li2—Ga1^iv^	−102.80 (13)	O6—B2—O4—Li2	0.4 (4)
Ga1—O4—Li2—Ga1^vi^	66.9 (3)	O6—B2—O5—Ga1^ii^	−101.3 (3)
Ga1—O4—Li2—Ga1^i^	−41.4 (3)	O6—B2—O5—Li1^x^	26.8 (3)
Ga1—O4—Li2—B1^viii^	95.37 (14)	O6—B2—O5—Li2^v^	127.1 (3)
Ga1—O4—Li2—B2^i^	−127.4 (3)	Li1^iii^—Ga1—O1—B1	−37.0 (2)
Ga1—O4—Li2—O1^i^	−12.7 (3)	Li1^iii^—Ga1—O1—Li2^v^	135.94 (19)
Ga1—O4—Li2—O2^viii^	95.0 (2)	Li1^iii^—Ga1—O4—B2	−105.3 (2)
Ga1—O4—Li2—O5^i^	−126.46 (19)	Li1^iii^—Ga1—O4—Li2	72.3 (2)
Ga1—O4—Li2—Li1^xi^	−166.36 (14)	Li1^viii^—B2—O4—Ga1	−140.43 (17)
Ga1—O4—Li2—Li3^xiii^	133.58 (18)	Li1^viii^—B2—O4—Li2	42.1 (3)
B2—O4—Li2—Ga1^iv^	75.0 (2)	Li1^viii^—B2—O5—Ga1^ii^	−149.04 (15)
B2—O4—Li2—Ga1^vi^	−115.2 (2)	Li1^viii^—B2—O5—Li1^x^	−21.0 (3)
B2—O4—Li2—Ga1^i^	136.4 (2)	Li1^viii^—B2—O5—Li2^v^	79.3 (3)
B2—O4—Li2—Ga1	177.8 (3)	Li1^viii^—B2—O6—Li1^xi^	−101.9 (3)
B2—O4—Li2—B1^viii^	−86.8 (2)	Li1^viii^—B2—O6—Li3	133.1 (3)
B2—O4—Li2—B2^i^	50.5 (4)	Li2^vi^—Ga1—O1—B1	76.7 (2)
B2—O4—Li2—O1^i^	165.1 (2)	Li2^v^—Ga1—O1—B1	−173.0 (3)
B2—O4—Li2—O2^viii^	−87.2 (3)	Li2^iv^—Ga1—O1—B1	−98.1 (2)
B2—O4—Li2—O5^i^	51.4 (3)	Li2—Ga1—O1—B1	142.1 (2)
B2—O4—Li2—Li1^xi^	11.5 (3)	Li2—Ga1—O1—Li2^v^	−44.96 (17)
B2—O4—Li2—Li3^xiii^	−48.6 (3)	Li2^vi^—Ga1—O1—Li2^v^	−110.28 (16)
O1—Ga1—O4—B2	42.2 (2)	Li2^iv^—Ga1—O1—Li2^v^	74.9 (2)
O1—Ga1—O4—Li2	−140.21 (19)	Li2—Ga1—O4—B2	−177.6 (3)
O1—B1—O2—Ga1^v^	−38.0 (3)	Li2^iv^—Ga1—O4—B2	−80.7 (2)
O1—B1—O2—Li2^viii^	86.5 (3)	Li2^vi^—Ga1—O4—B2	130.6 (2)
O1—B1—O2—Li3^ix^	−166.5 (2)	Li2^v^—Ga1—O4—B2	33.0 (2)
O1—B1—O3—Li1	−143.3 (3)	Li2^iv^—Ga1—O4—Li2	96.9 (2)
O1—B1—O3—Li3^ii^	89.1 (3)	Li2^vi^—Ga1—O4—Li2	−51.7 (2)
O1—B1—O3—Li3^vii^	−8.1 (5)	Li2^v^—Ga1—O4—Li2	−149.4 (3)
O2^i^—Ga1—O1—B1	48.3 (2)	Li2^viii^—B1—O1—Ga1	−145.49 (15)
O2^i^—Ga1—O1—Li2^v^	−138.71 (18)	Li2^viii^—B1—O1—Li2^v^	43.1 (4)
O2^i^—Ga1—O4—B2	166.3 (2)	Li2^viii^—B1—O2—Ga1^v^	−124.5 (2)
O2^i^—Ga1—O4—Li2	−16.1 (2)	Li2^viii^—B1—O2—Li3^ix^	107.0 (2)
O2—B1—O1—Ga1	165.44 (16)	Li2^viii^—B1—O3—Li1	−14.5 (3)
O2—B1—O1—Li2^v^	−5.9 (4)	Li2^viii^—B1—O3—Li3^vii^	120.7 (3)
O2—B1—O3—Li1	38.4 (4)	Li2^viii^—B1—O3—Li3^ii^	−142.1 (2)
O2—B1—O3—Li3^ii^	−89.2 (3)	Li2^v^—B2—O4—Ga1	−34.6 (2)
O2—B1—O3—Li3^vii^	173.6 (3)	Li2^v^—B2—O4—Li2	147.9 (3)
O3—B1—O1—Ga1	−13.0 (3)	Li2^v^—B2—O5—Ga1^ii^	131.7 (2)
O3—B1—O1—Li2^v^	175.7 (3)	Li2^v^—B2—O5—Li1^x^	−100.3 (3)
O3—B1—O2—Ga1^v^	140.4 (2)	Li2^v^—B2—O6—Li1^xi^	−151.7 (3)
O3—B1—O2—Li2^viii^	−95.1 (3)	Li2^v^—B2—O6—Li1^viii^	−49.8 (4)
O3—B1—O2—Li3^ix^	12.0 (3)	Li2^v^—B2—O6—Li3	83.2 (4)
O4—Ga1—O1—B1	171.88 (18)	Li3^vii^—Ga1—O1—B1	16.7 (2)
O4—Ga1—O1—Li2^v^	−15.1 (2)	Li3^vii^—Ga1—O1—Li2^v^	−170.3 (2)
O4—B2—O5—Ga1^ii^	80.4 (2)	Li3^vii^—Ga1—O4—B2	162.9 (3)
O4—B2—O5—Li1^x^	−151.6 (2)	Li3^vii^—Ga1—O4—Li2	−19.4 (3)
O4—B2—O5—Li2^v^	−51.3 (3)	Li3^ii^—B1—O1—Ga1	33.9 (2)
O4—B2—O6—Li1^viii^	78.0 (3)	Li3^ii^—B1—O1—Li2^v^	−137.4 (3)
O4—B2—O6—Li1^xi^	−23.9 (4)	Li3^ii^—B1—O2—Ga1^v^	94.3 (2)
O4—B2—O6—Li3	−149.0 (3)	Li3^ii^—B1—O2—Li2^viii^	−141.2 (2)
O5^ii^—Ga1—O1—B1	−70.85 (19)	Li3^ii^—B1—O2—Li3^ix^	−34.2 (3)
O5^ii^—Ga1—O1—Li2^v^	102.13 (18)	Li3^ii^—B1—O3—Li1	127.6 (3)
O5^ii^—Ga1—O4—B2	−69.7 (2)	Li3^ii^—B1—O3—Li3^vii^	−97.2 (3)
O5^ii^—Ga1—O4—Li2	107.94 (19)	Li3—B2—O4—Ga1	139.9 (3)
O5—B2—O4—Ga1	−3.7 (3)	Li3—B2—O4—Li2	−37.5 (5)
O5—B2—O4—Li2	178.8 (2)	Li3—B2—O5—Ga1^ii^	−81.93 (16)
O5—B2—O6—Li1^viii^	−100.3 (3)	Li3—B2—O5—Li1^x^	46.1 (2)
O5—B2—O6—Li1^xi^	157.8 (3)	Li3—B2—O5—Li2^v^	146.4 (2)
O5—B2—O6—Li3	32.7 (4)	Li3—B2—O6—Li1^viii^	−133.1 (3)
O6—B2—O4—Ga1	177.91 (18)	Li3—B2—O6—Li1^xi^	125.1 (4)

Symmetry codes: (i) *x*+1, *y*, *z*; (ii) −*x*+1, −*y*+1, −*z*+1; (iii) −*x*+1, −*y*, −*z*+2; (iv) −*x*+2, −*y*+1, −*z*+1; (v) *x*−1, *y*, *z*; (vi) −*x*+2, −*y*, −*z*+1; (vii) *x*, *y*−1, *z*+1; (viii) −*x*+1, −*y*, −*z*+1; (ix) *x*−1, *y*−1, *z*+1; (x) *x*, *y*+1, *z*−1; (xi) *x*+1, *y*+1, *z*−1; (xii) −*x*, −*y*−1, −*z*+2; (xiii) −*x*+2, −*y*+1, −*z*; (xiv) −*x*+1, −*y*+2, −*z*.
